# Giant colloidal silver crystals for low-loss linear and nonlinear plasmonics

**DOI:** 10.1038/ncomms8734

**Published:** 2015-07-15

**Authors:** Chun-Yuan Wang, Hung-Ying Chen, Liuyang Sun, Wei-Liang Chen, Yu-Ming Chang, Hyeyoung Ahn, Xiaoqin Li, Shangjr Gwo

**Affiliations:** 1 Department of Physics, National Tsing-Hua University, Hsinchu 30013, Taiwan.; 2 Department of Physics, The University of Texas at Austin, Austin, Texas 78712, USA.; 3 Center for Condensed Matter Sciences, National Taiwan University, Taipei 10617, Taiwan.; 4 Department of Photonics, National Chiao-Tung University, Hsinchu 30010, Taiwan.; 5 National Synchrotron Radiation Research Center, Hsinchu 30076, Taiwan.

## Abstract

The development of ultrasmooth, macroscopic-sized silver (Ag) crystals exhibiting reduced losses is critical to fully characterize the ultimate performance of Ag as a plasmonic material, and to enable cascaded and integrated plasmonic devices. Here we demonstrate the growth of single-crystal Ag plates with millimetre lateral sizes for linear and nonlinear plasmonic applications. Using these Ag crystals, surface plasmon polariton propagation lengths beyond 100 μm in the red wavelength region are measured. These lengths exceed the predicted values using the widely cited Johnson and Christy data. Furthermore, they allow the fabrication of highly reproducible plasmonic nanostructures by focused ion beam milling. We have designed and fabricated double-resonant nanogroove arrays using these crystals for spatially uniform and spectrally tunable second-harmonic generation. In conventional ‘hot-spot'-based nonlinear processes such as surface-enhanced Raman scattering and second-harmonic generation, strong enhancement can only occur in random, localized regions. In contrast, our approach enables uniform nonlinear signal generation over a large area.

## Abstract (web summary)

The typical small sizes of silver microcrystals prevent the full characterization of fundamental plasmonic properties and limits applications. Here, Wang *et al*. report the synthesis of large colloidal silver crystals with superior nonlinear properties and surface plasmon polariton propagation lengths beyond 100 μm.

## Graphical abstract

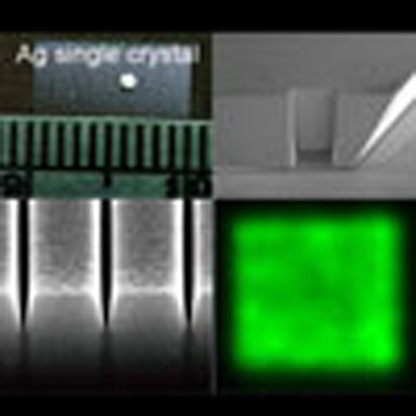

Plasmonic nanostructures and metamaterials offer unique possibilities for manipulating and amplifying linear and nonlinear optical processes at subwavelength scales^1^,^2^,^3^,^4^,^5^,^6^,^7^,^8^,^9^,^10^,^11^. At optical frequencies, silver (Ag) is the preferred plasmonic material owing to its lowest intrinsic loss among all metals^12^,^13^,^14^,^15^,^16^. However, additional scattering losses originated from grain boundaries and surface roughness limit the performance of polycrystalline Ag plasmonic structures prepared by conventional techniques. Recognizing the limitations of polycrystalline Ag prepared by traditional deposition methods with largely variable loss, researchers have devoted extensive effort to develop new chemical synthesis^13^ and film growth^15^,^16^ techniques to obtain single-crystalline Ag structures. For example, epitaxial silver films with atomic smoothness^16^ have been developed and shown to greatly enhance the performance of plasmonic nanolasers^17^. The growth process of atomically smooth epitaxial Ag films is, however, quite tedious owing to the slow iterative two-step process consisting of low temperature deposition and room temperature annealing in ultrahigh vacuum^16^. As a result, only films with limited thicknesses (typically, tens of nm) can be prepared. A simple yet effective method, template stripping, has been applied to produce Ag films with atomically smooth top surface and led to a longer surface plasmon polariton (SPP) propagation length at the film/air interface, which is ∼30 μm in the red wavelength region^12^. Nevertheless, a large number of grain boundaries still exist inside the stripped films, which cause additional losses and reduce the SPP propagation length. Alternatively, colloidal Ag microcrystals have been grown using chemical synthetic methods^13^,^18^,^19^,^20^. However, the small sizes of such crystals (typically, up to tens of μm in lateral dimension) still prevent the full characterization of fundamental plasmonic properties and limit their practical applications.

Here we report the synthesis of giant colloidal Ag single crystals with millimetre lateral size and tens of microns in thickness. Using these crystals, we achieve SPP propagation lengths beyond 100 μm in the red wavelength region. These values even exceed the predicted propagation length using the widely cited optical constants by Johnson and Christy (JC)^21^, and others^22^,^23^, which have been extensively used to design and model plasmonic devices and metamaterials. Furthermore, we have designed and fabricated double-resonant ‘V'-shaped nanogroove arrays for efficient second-harmonic generation (SHG). Conventional nonlinear optics requires phase matching and long coherent length, which are not easy to be realized at the nanoscale. Instead of strict phase-matching conditions, nanostructures based on plasmonic metals (Ag, Au and so on) facilitate the formation of subwavelength hot zones to enhance the nonlinear processes. Furthermore, additional nonlinear enhancement can be achieved by embedding other nonlinear media at the hot zones^8^. Compared with surface-enhanced nonlinear signal generation based on local hot spots^24^,^25^, our method using the single-crystalline material platform enables spatially uniform, spectrally narrow and tunable nonlinear signal generation over a larger area. We expect that the results presented here can stimulate future progress in both plasmonic material synthesis and applications, particularly in the areas of integrated linear and nonlinear plasmonic devices.

## Results

### Synthesis and characterization of Ag plates

**Figure 1:**
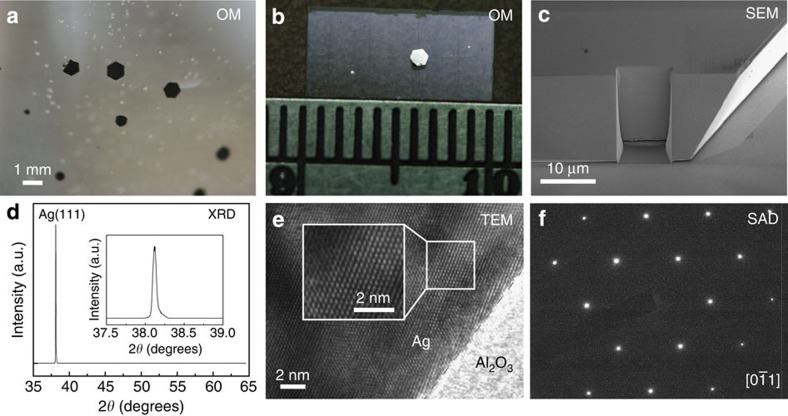
Structural properties of giant colloidal silver single crystals. (**a**,**b**) Optical microscopy (OM) images of colloidal Ag single crystals in solution and on an indium-tin-oxide-coated glass substrate, respectively. (**c**) Scanning electron microscopy (SEM) image of Ag crystal. Part of the Ag crystal sidewall was modified by FIB milling. (**d**) XRD wide-scan pattern shows only the Ag (111) peak. The FWHM of the Ag (111) peak determined by high-resolution XRD is <0.05°, which is close to the instrument limit. (**e**) High-resolution TEM image of Ag crystal. The TEM sample was prepared by FIB milling from the top facet of Ag crystal. (**f**) Selected area diffraction pattern obtained from the TEM image area shown in **e**.

The giant single-crystalline Ag plates are synthesized at room temperature using a modified platinum-catalyzed, ammonium hydroxide (NH_4_OH)-controlled polyol reduction method^13^,^18^. To enlarge the grown crystal size, we increase the concentration of NH_4_OH for a drastically decreased reaction rate (see details in Methods section). Here NH_4_OH plays the role of stabilizer in the reduction process. A supersaturated solution of NH_4_OH used here results in the formation of Ag(NH_3_)_*n*_^+^ complex with a large *n*, and thus decreasing the Ag reduction rate greatly. In the polyol process, controlling the nucleation and growth steps in the reaction media is critical for controlling Ag crystal size and shape. The reaction time in this work takes a few days instead of a few hours as the previous report^18^. During the growth process, the reaction media remain clear, and after reaction giant colloidal Ag plates with millimetre-scale lateral sizes can be found on the bottom or sidewall of the glass container, as shown in Fig. 1a.

We conducted a number of experiments to characterize the structural properties of the single-crystalline Ag plates. Optical microscopy (Fig. 1a,b) and scanning electron microscopy (Fig. 1c) images clearly show that these crystals have millimetre-scale lateral size and thickness of tens of microns, which are the largest colloidal Ag single crystals ever reported in the literature^13^,^18^,^19^,^20^. In Fig. 1c, we also show the ultrasmooth finish of focused ion beam (FIB)-milled edges on the Ag crystal sidewall. Previously, it has also been demonstrated that reproducible and precise (high-fidelity) FIB nanofabrication of plasmonic structures can be realized on single-crystalline gold (Au) microflakes^26^. In comparison with Au, surface passivation is critical for the fabrication of Ag plasmonic structures. To this end, only fresh Ag plates were used for characterization and fabrication, and a 5-nm-thick Al_2_O_3_ conformal coating layer was immediately grown after FIB milling by using atomic layer deposition to protect the Ag structures against surface degradation (mainly sulfidation)^13^ in ambient environment. Furthermore, we confirmed the crystalline quality by using X-ray diffraction (XRD) and high-resolution transmission electron microscopy (TEM). Only the Ag (111) peak was observed in the XRD data, and its full-width at half-maximum (FWHM) is instrument limited to be <0.05° (Fig. 1d). This narrow peak width in XRD suggests that the crystallinity of grown crystals is greatly improved in comparison with previous works that have typically demonstrated XRD peaks of a 0.3–0.45° width^15^,^20^,^27^, indicating much bigger single-crystal domain sizes in our crystals. In addition, these silver crystals were confirmed to have a face-centered cubic structure (Fig. 1e,f) by using the selected area diffraction pattern in TEM. The surface smoothness of Ag crystal was also examined by atomic force microscopy shown in Supplementary Fig. 1 and Supplementary Note 1. The Ag crystal surface has a root mean square roughness of 0.5 nm over 1 × 1 μm^2^ area, which is comparable to an epitaxial Ag film^16^.

### SPP propagation length measurements

**Figure 2:**
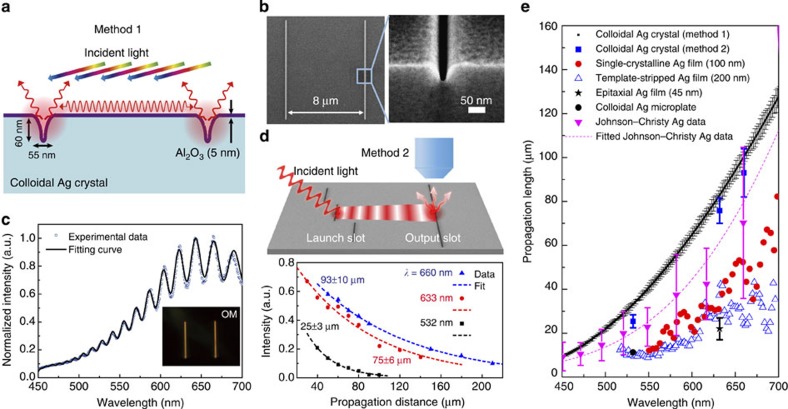
SPP propagation lengths on giant colloidal silver single crystals. (**a**) Schematic of the WLI setup for measuring the SPP propagation lengths on the Ag crystal. The incident angle of the halogen white light is about 75–80°. (**b**) Top-view and tilted-view SEM images of the FIB-milled nanogrooves. (**c**) The SPP scattering signal collected by an optical microscope. The inset shows the scattering image from the double-groove structure. (**d**) Schematic and experimental results of the DSI method for measuring the SPP propagation lengths. (**e**) SPP propagation lengths measured with both WLI: black squares and DSI: blue squares methods, in comparison with those reported for different Ag samples in the visible range (red circles: ref. 15, blue open triangles: ref. 12, black stars: ref. 16, black circles: ref. 20, pink inverted triangles: ref. 21). The error bars shown for method 1 represent s.d. of measurement values from several double-slit structures (see Supplementary Fig. 3). The error bars shown for method 2 are s.d. in **d**. The predicted propagation length using the JC Ag data were calculated by the eigenmode method for an air/5 nm-Al_2_O_3_/Ag planar structure, and the error bars are from the JC data (ref. 21).

The low-loss linear optical property of single-crystalline Ag plate is demonstrated via the SPP propagation length measurements, which confirm the longest SPP propagation lengths in the visible frequency range to date. We measured SPP propagation lengths using two different methods: white-light interference (WLI, Fig. 2a–c) and direct scattered intensity (DSI, Fig. 2d) from a series of output coupling slots. The interpretation of the DSI method is rather straightforward^12^,^15^,^16^. However, DSI is a tedious method that only provided SPP propagation lengths at a few available laser wavelengths. In contrast, the WLI method allows us to extract propagation lengths from continuous wavelengths in the range of 450–700 nm with the assistance of modelling and simulations (see Supplementary Note 2a). In our study, the consistency of the results from these two completely different methods confirms the validity of measured propagation lengths.

For the WLI experiments, double nanogrooves of 10 μm length were milled by FIB (Fig. 2b) using a freshly synthesized Ag plate. We used a halogen white light to excite surface plasmons in the nanogrooves with an incident angle of 75–80° (see also Supplementary Fig. 2). The surface plasmon modes excited in the nanogrooves can be partially coupled out to launch SPPs propagating along the Ag film and partially coupled to radiation detected in the far field. The propagating SPPs then make several round trips between the two nanogrooves and eventually couple to the far-field radiation. The radiative field was collected through a microscope objective ( × 100, numerical aperture N.A.=0.8), and the spectrum exhibited a clear Fabry–Pérot interference pattern as a function of wavelength. We adopted a simple model to fit the experimental data (Fig. 2c) and the fitting results provide both the real and imaginary parts of the SPP wavenumber, that is, Re(*k*_SPP_) and Im(*k*_SPP_). We then used the fitted Re(*k*_SPP_) and Im(*k*_SPP_) to acquire the effective index *n*_eff_=Re(*k*_SPP_)·*λ*/2*π* (Supplementary Fig. 4) and the propagation length *L*_SPP_=1/(2·Im(*k*_SPP_); Fig. 2e). These data points are averaged measurement values from several double-slit structures (see Supplementary Fig. 3) as explained in detail in Supplementary Note 2a.

To ensure that the SPP propagation length reported above is independent of the model and fitting procedure, we used a completely different and a simpler method, DSI, to check SPP propagation length at a few discrete wavelengths. For the DSI experiments, a long groove of 250 μm length and a series of short grooves of 25 μm length with increasing distance away from the long groove were milled by using FIB. SPPs were launched from individual short groove illuminated by an oblique incident laser beam, and detected in the far field after scattering at the output long groove (Fig. 2d). The intensity of the scattered laser from the output groove was measured as a function of the distance between the launching and output grooves and fitted with an exponential function. The propagation lengths measured at three different wavelengths (532, 632 and 660 nm) were plotted in Fig. 2d. The extracted propagation distances (25±3, 75±6 and 93±10 μm) agree well with those from the WLI measurements. The small discrepancy arises from the fact that a larger sample area is necessary using the DSI method. Thus, there is increased scattering loss from possible surface imperfection or contamination.

### SHG measurements

Following the characterization of linear optical properties, we now turn to the enhanced nonlinear optical properties enabled by these giant Ag crystals, where improvements due to low material loss and high-fidelity nanofabrication are expected to be even more dramatic as nonlinearity is proportional to higher orders of the local field enhancement^3^,^4^,^5^,^6^,^7^,^8^,^9^,^10^. In particular, we focus on the widely investigated SHG on the Ag surface^28^,^29^. Previous experiments on nonlinear plasmonics were based on nanoroughened films^25^ or nanoantenna arrays^5^,^6^,^7^,^8^,^9^ fabricated on thermally evaporated or sputtered noble metal films, where strong nonlinear signals are only generated at local hot spots with a random spatial distribution or in a small fraction of fabricated nanodevices arrays^6^,^9^, despite of the recent advance in helium-ion-based FIB milling^9^. As a result, the uncontrollability and low yield of working devices make large-scale integration of plasmonic devices impractical.

**Figure 3:**
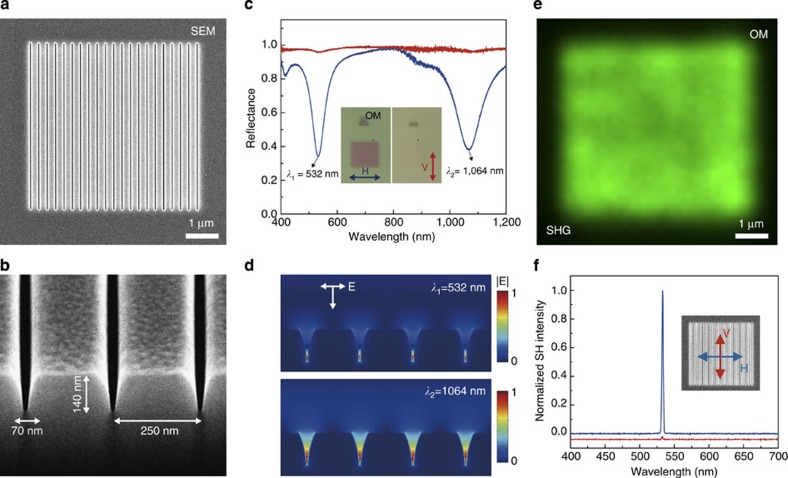
Spatially uniform SHG emission from a double-resonant plasmonic nanogroove array. (**a**,**b**) SEM images of the FIB-milled, V-shaped nanogroove array structure, which has double plasmonic resonances at *λ*_1_=532 nm and *λ*_2_=1,064 nm. (**c**) Optical reflectance spectra at normal incidence show strong dependence on the incident light polarization. The blue curve is measured with a horizontal (H) polarized white light (electric field perpendicular to nanogroove). In contrast, the vertical (V) polarized white light (electric field parallel to nanogroove) has reflectance close to unity (red curve). The insets show the OM images. (**d**) Finite-difference time-domain (FDTD) simulations of near-field distributions at double plasmonic resonances excited by an H-polarized normal-incident light. (**e**) OM image of spatially uniform SHG emission (532 nm) mapped by scanning a focused laser beam (H-polarized, pulsed, 1,064 nm). (**f**) The SHG emission at 532 nm is H-polarized with a large polarization ratio.

Here we show the unique capability to produce strongly enhanced and highly uniform nonlinear SHG over a large fabricated area. For this purpose, we designed a V-nanogroove array and created the pattern by FIB milling (Fig. 3a,b). The width, depth and pitch of nanogrooves are designed to support double plasmon resonances at *λ*_1_=532 nm (SHG) and *λ*_2_=1,064 nm (fundamental). The double resonance concept has been applied before for enhanced SHG using plasmonic nanoantenna structures^30^,^31^. In comparison, the key advantages of the double-resonant nanogroove array used here are large and uniform emission area, narrow spectrum and wavelength tunable SHG.

The design principle is that the V-nanogroove acts as a plasmonic nanocavity that strongly confines plasmons along the depth direction via standing waves of counter-propagating SPPs reflected at the bottom and opening of the groove^32^,^33^. The resonant cavity modes can be precisely controlled by the width and depth of nanogroove (also affected by coupling between grooves in the case of nanogroove array^32^). Moreover, the nanogroove shape can focus the SHG field at the bottom of the V-shaped groove to yield a large field enhancement^34^. The grating structure is adopted here to achieve large-area SHG emission and additional field enhancement by plasmonic near-field coupling of nanogrooves separated by a subwavelength distance^32^. In Supplementary Information, we show that a nanogroove array with 250 nm pitch can result in a threefold field intensity enhancement at the SHG wavelength compared with a single nanogroove (Supplementary Fig. 6 and Supplementary Note 3a).

The double resonance can be clearly observed by polarization-dependent reflectance measurements. In the reflectance spectra shown in Fig. 3c, the blue (red) curve exhibits strong (minimal) absorption at the designed resonant wavelengths when the incident polarization is perpendicular (parallel) to the nanogrooves (see Supplementary Note 3b for detail). This polarization dependence is consistent with the simulation results shown in Fig. 3d. The SHG emission signal at 532 nm excited by an H-polarized incident laser beam at 1,064 nm (that is, the incident electric field is perpendicular to the nanogrooves) was found to be H-polarized (Fig. 3e,f). The strong emission polarization (polarization contrast as large as 1:70) illustrates again that the simulated plasmonic modes (Fig. 3d) are responsible for the strongly enhanced SHG signal. We have also confirmed that the SHG signal shows a quadratic dependence on the excitation intensity (Supplementary Fig. 7 and Supplementary Note 3c,d).

## Discussion

The SPP propagation lengths measured from our Ag crystals exceed those in all previous studies including the predicted values from JC data. Because of the elimination of grain boundaries inside the crystals, the SPP propagation lengths measured on our sample is longer than those measured from a template-stripped film as previously reported^12^. While epitaxially grown Ag films should exhibit similar intrinsic loss to our Ag single crystals, an additional leaky mode reduced the SPP propagation length measured from a 45-nm-thick epitaxial film^16^. In samples >100 nm thick^12^,^15^,^20^,^21^,^22^,^23^, the propagation lengths no longer depend on the thickness (see Supplementary Fig. 5 and Supplementary Note 2c for detail). This exceptionally long SPP propagation distance suggests that the colloidal Ag crystal has a lower loss than the value described by the widely cited JC permittivity data^21^, which represent the lowest loss of Ag in the past 40 years. Until now, most theoretical studies tend to use the JC data to model plasmonic phenomena, while the typical experimental results show more losses than those predicted by the JC data.

**Figure 4:**
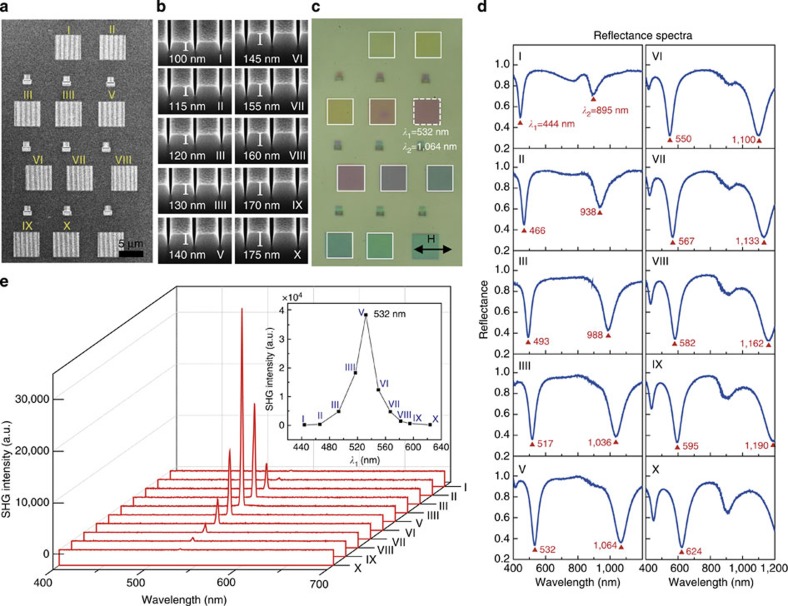
SHG emissions from a series of double-resonant plasmonic structures. (**a**,**b**) SEM images of a series of nanogroove array structures with different nanogroove depths. (**c**) Corresponding reflection OM images of nanogroove structures acquired with an H-polarized, normal incident white light. The reflection colours of the OM images depend on the spectral positions of plasmonic resonances. (**d**) Corresponding white-light reflectance spectra of reflective OM images shown in **c**. (**e**) Corresponding SHG spectra of nanogroove structures shown in **a** excited using a 1,064 nm pulsed laser. The SHG signal is maximized when the excitation laser wavelength and the SHG emission wavelength match with the double-resonant plasmonic modes supported by the nanogroove array. The inset shows the variation of SHG peak intensity at 532 nm with respect to the plasmonic resonances (*λ*_1_) of different nanogroove structures. According to this plot, a 1.5-nm variation of nanogroove depth from the optimized depth can lead to about 10% decrease in the SHG intensity.

For the applications in nonlinear plasmonics, there are several unique advantages of the SHG process demonstrated here. First of all, we achieve large-area SHG over the whole patterned area (5 × 5 μm^2^), in contrast to random, localized regions in conventional ‘hot-spot'-based nonlinear plasmonic processes^5^,^6^,^7^,^8^,^9^,^24^,^25^. Because of the highly uniform enhancement in a large ‘hot area', one may further increase the nonlinear conversion efficiency by filling the V-nanogroove with a nonlinear medium^8^. The s.d. of the SHG intensity is <10% over the whole patterned area, visible from the optical image shown in Fig. 3e (detailed image analysis is shown in Supplementary Fig. 8). A small groove depth variation of ∼1.5 nm from the optimal groove depth, assuming constant groove width and pitch, can results in a 10% SHG intensity drop from the optimized SHG intensity as verified by results presented in Fig. 4. It should be noted that this depth fluctuation is already close to the instrumental limitation of gallium-ion-based FIB milling.

Second, the plasmonic resonances of nanogroove array are spectrally narrow with an FWHM width of ∼50 nm for the peak at 532 nm and ∼100 nm for the peak at 1,064 nm. This narrow spectrum originates from localized cavity modes confined in nanogrooves and plasmonic coupling with neighbouring nanogrooves^32^, as well as delocalized grating modes propagating on the surface^35^. Spectrally narrow nonlinear signal generation opens the possibility for wavelength multiplexing applications, which require well-defined spectral windows. Third, the designed resonant wavelengths can be precisely and continuously tuned by changing the width, depth and pitch of the nanogroove array. We have demonstrated tuning of the plasmonic resonant wavelengths over a wide wavelength range of 180 nm (*λ*_1_, which is tuned to be resonant with the SHG emission wavelength) by precisely controlling the milling depth of the nanogrooves from 100 to 175 nm (step sizes: ∼5–15 nm, see Fig. 4a–d and Supplementary Fig. 9). The corresponding SHG spectra from a series of nanogroove array structures (marked from I to X in Fig. 4a) taken with the same pulsed excitation laser at 1,064 nm are shown in Fig. 4e. These spectra illustrate a strong reduction in the SHG intensity from detuned nanogroove arrays (up to 200 × reduction for the case of nonresonant structure I versus resonant structure V). Finally, the design adopted here allows that both the fundamental and second-harmonic fields to be greatly enhanced, leading to >2,000 times stronger SHG in comparison with that from the unpatterned surface (see Supplementary Fig. 10). All the desirable features for SHG generation (large-area SHG generation, high spatial uniformity and tunable narrow resonances) can only be realized by using single-crystalline Ag plates. The absence of surface roughness and granularity in these Ag crystals enables us to create high-fidelity nanogrooves with well-controlled dimensions, which are limited only by the FIB instrument.

In summary, the giant colloidal Ag crystals reported here represent a new plasmonic material platform for low-loss linear and nonlinear plasmonic applications. The resulting long SPP propagation length in the linear regime can open up new possibilities of fabricating integrated plasmonic devices. These crystals also enable strongly enhanced nonlinear signal generation uniformly over a large patterned area, a testimony for their unparalleled material properties and enormous potential for applications in nonlinear plasmonics. In addition, one can deposit semiconductor nanostructures or molecules on these crystals for applications in plasmonic nanolasers and plasmon-facilitated molecular energy transfer.

## Methods

### Synthesis of giant colloidal silver single crystals

The giant single-crystalline Ag plate were synthesized by using a platinum (Pt)-nanoparticle-catalyzed and ammonium hydroxide (NH_3_OH)-controlled polyol reduction method^13^,^18^. First, we prepared 0.17 M silver nitrate 15 ml (AgNO_3_, Sigma-Aldrich) ethylene glycol solution (EG, Sigma-Aldrich) as the precursor and added excess of ammonium hydroxide (NH_4_OH, 8 M, 1.85 ml, J.T. Baker) solution in a glass vial. Second, a small amount of polyvinylpyrrolidone (*M*_w_=55,000, 0.5 g, Sigma-Aldrich) was added to the solution and mixed uniformly by magnetic stirring. Here polyvinylpyrrolidone is the dispersing and capping agent in the reduction process^13^. Then, chloroplatinic acid hydrate (H_2_PtCl_6_·*x*H_2_O, 0.02 M, 0.54 ml, Sigma-Aldrich) was added to the solution to form Pt nanoparticles. Finally, a hydrogen peroxide (H_2_O_2_, 33%, 1.3 ml, Sigma-Aldrich) solution was injected into the mixture solution as the Ag reducing agent. The total reaction time is about 5 days at room temperature. After the reaction was completed, deionized water and acetone were used to purge silver single crystals. Previously, the typical size of silver flakes grown by this method is few tens of microns^18^. To growth giant silver crystals, the important control factor is the concentration of NH_4_OH, which plays the role of a stabilizer in the reaction process. A supersaturated solution of NH_4_OH would make large Ag(NH_3_)_*n*_^+^ complexes more stable and result in the decrease of the Ag reduction rate.

### XRD measurement

High-resolution XRD wide-scan patterns were acquired by a commercial XRD system (HUBER 6-circle diffractometer 424–512.51). The 2*θ* resolution is about 0.05° with a detector slit of 0.5 mm.

### Fabrication of nanogroove structures

All of the nanogroove structures on the Ag single crystals were fabricated by using a FIB system (FEI Helios Nanolab 600i). These structures were milled at a 30-kV acceleration voltage and an ion beam current of 1.1 pA.

## Additional information

**How to cite this article:** Wang, C. Y. *et al*. Giant colloidal silver crystals for low-loss linear and nonlinear plasmonics. *Nat. Commun.* 6:7734 doi: 10.1038/ncomms8734 (2015).

## Supplementary Material

Supplementary Information Supplementary Figures 1-10 and Supplementary Notes 1-3

## References

[b1] Barnes W. L., Dereux A. & Ebbesen T. W. Surface plasmon subwavelength optics. Nature 424, 824–830 (2003). 12917696 10.1038/nature01937

[b2] Schuller J. A. . Plasmonics for extreme light concentration and manipulation. Nat. Mater. 9, 193–204 (2010). 20168343 10.1038/nmat2630

[b3] Kauranen M. & Zayats A. V. Nonlinear plasmonics. Nat. Photonics 6, 737–748 (2012).

[b4] Bouhelier A., Beversluis M., Hartschuh A. & Novotny L. Near-field second-harmonic generation induced by local field enhancement. Phys. Rev. Lett. 90, 013903 (2003). 12570612 10.1103/PhysRevLett.90.013903

[b5] Mühlschlegel P., Eisler H.-J., Martin O. J. F., Hecht B. & Pohl D. W. Resonant optical antennas. Science 308, 1607–1609 (2005). 15947182 10.1126/science.1111886

[b6] Schuck P. J., Fromm D. P., Sundaramurthy A., Kino G. S. & Moerner W. E. Improving the mismatch between light and nanoscale objects with gold bowtie nanoantennas. Phys. Rev. Lett. 94, 017402 (2005). 15698131 10.1103/PhysRevLett.94.017402

[b7] Kim S. . High-harmonic generation by resonant plasmon field enhancement. Nature 453, 757–760 (2008). 18528390 10.1038/nature07012

[b8] Aouani H., Rahmani M., Navarro-Cia M. & Maier S. A. Third-harmonic-upconversion enhancement from a single semiconductor nanoparticle coupled to a plasmonic antenna. Nat. Nanotechnol. 9, 290–294 (2014). 24608232 10.1038/nnano.2014.27

[b9] Kollmann H. . Toward plasmonics with nanometer precision: nonlinear optics of helium-ion milled gold nanoantennas. Nano Lett. 14, 4778–4784 (2014). 25051422 10.1021/nl5019589

[b10] Zhang Y., Grady N. K., Ayala-Orozco C. & Halas N. J. Three-dimensional nanostructures as highly efficient generators of second harmonic light. Nano Lett. 11, 5519–5523 (2011). 22043857 10.1021/nl2033602

[b11] Suchowski H. . Phase mismatch–free nonlinear propagation in optical zero-index materials. Science 342, 1223–1226 (2013). 24311687 10.1126/science.1244303

[b12] Nagpal P., Lindquist N. C., Oh S.-H. & Norris D. J. Ultrasmooth patterned metals for plasmonics and metamaterials. Science 325, 594–597 (2009). 19644116 10.1126/science.1174655

[b13] Rycenga M. . Controlling the synthesis and assembly of silver nanostructures for plasmonic applications. Chem. Rev. 111, 3669–3712 (2011). 21395318 10.1021/cr100275d PMC3110991

[b14] Boltasseva A. & Atwater H. A. Low-loss plasmonic metamaterials. Science 331, 290–291 (2011). 21252335 10.1126/science.1198258

[b15] Park J. H. . Single-crystalline silver films for plasmonics. Adv. Mater. 24, 3988–3992 (2012). 22700389 10.1002/adma.201200812

[b16] Wu Y. . Intrinsic optical properties and enhanced plasmonic response of epitaxial silver. Adv. Mater. 26, 6106–6110 (2014). 24923858 10.1002/adma.201401474

[b17] Lu Y. J. . Plasmonic nanolaser using epitaxially grown silver film. Science 337, 450–453 (2012). 22837524 10.1126/science.1223504

[b18] Liang H. Z. . Mechanism for the formation of flake silver powder synthesized by chemical reduction in ethylene glycol. Acta Phys.Chim. Sin. 19, 150–153 (2003).

[b19] Ditlbacher H. . Silver nanowires as surface plasmon resonators. Phys. Rev. Lett. 95, 257403 (2005). 16384506 10.1103/PhysRevLett.95.257403

[b20] Chang C. W. . HNO_3_-assisted polyol synthesis of ultralarge single-crystalline Ag microplates and their far propagation length of surface plasmon polariton. ACS Appl. Mater. Interfaces 6, 11791–11798 (2014). 24987801 10.1021/am502549d

[b21] Johnson P. B. & Christy R. W. Optical constants of the noble metals. Phys. Rev. B 6, 4370–4379 (1972).

[b22] Lynch D. W. & Hunter W. R. in Handbook of Optical Constants of Solids ed. Palik E. D.) Vol. I, 350–357Academic Press (1998).

[b23] Lide D. R. (ed.) CRC Handbook of Chemistry and Physics CRC Press (2008).

[b24] Jeanmaire D. L. & Van Duyne R. P. Surface Raman spectroelectrochemistry part I. heterocyclic, aromatic, and aliphatic amines on the anodized silver electrode. J. Electroanal.Chem. 84, 1–20 (1977).

[b25] Chen C. K., de Castro A. R. B. & Shen Y. R. Surface-enhanced second-harmonic generation. Phys. Rev. Lett. 46, 145–148 (1981).

[b26] Huang J.-S. . Atomically flat single-crystalline gold nanostructures for plasmonic nanocircuitry. Nat. Commun. 1, 150 (2010). 21267000 10.1038/ncomms1143

[b27] Chou B.-T., Lin S.-D., Huang B.-H. & Lu T.-C. Single-crystalline silver film grown on Si (100) substrate by using electron-gun evaporation and thermal treatment. J. Vac. Sci. Technol. B 32, 031209 (2014).

[b28] Simon H. J., Mitchell D. E. & Watson J. G. Optical second-harmonic generation with surface plasmons in silver films. Phys. Rev. Lett. 33, 1531–1534 (1974).

[b29] Grosse N. B., Heckmann J. & Woggon U. Nonlinear plasmon-photon interaction resolved by k-space spectroscopy. Phys. Rev. Lett. 108, 136802 (2012). 22540719 10.1103/PhysRevLett.108.136802

[b30] Park S., Hahn J. W. & Lee J. Y. Doubly resonant metallic nanostructure for high conversion efficiency of second harmonic generation. Opt. Express 20, 4856–4870 (2012). 22418292 10.1364/OE.20.004856

[b31] Thyagarajan K., Rivier S., Lovera A. & Martin O. J. F. Enhanced second-harmonic generation from double resonant plasmonic antennae. Opt. Express 20, 12860–12865 (2012). 22714312 10.1364/OE.20.012860

[b32] Tan W.-C., Preist T. W., Sambles J. R. & Wanstall N. P. Flat surface-plasmon-polariton bands and resonant optical absorption on short-pitch metal gratings. Phys. Rev. B 59, 12661–12666 (1999).

[b33] Søndergaard T. . Resonant plasmon nanofocusing by closed tapered gaps. Nano Lett. 10, 291–295 (2010). 20028028 10.1021/nl903563e

[b34] Stockman M. I. Nanofocusing of optical energy in tapered plasmonic waveguides. Phys. Rev. Lett. 93, 137404 (2004). 15524758 10.1103/PhysRevLett.93.137404

[b35] Genevet P. . Large enhancement of nonlinear optical phenomena by plasmonic nanocavity gratings. Nano Lett. 10, 4880–4883 (2010). 21047115 10.1021/nl102747v

