# Supplementary material for: Giant colloidal silver crystals for low-loss linear and nonlinear plasmonics
Source: Nat Commun. 2015 Jul 15;6:7734. doi: 10.1038/ncomms8734 (PMC4518272; doi:10.1038/ncomms8734)
Supplement: Supplementary Information — Supplementary Figures 1-10 and Supplementary Notes 1-3 [file ncomms8734-s1.pdf]

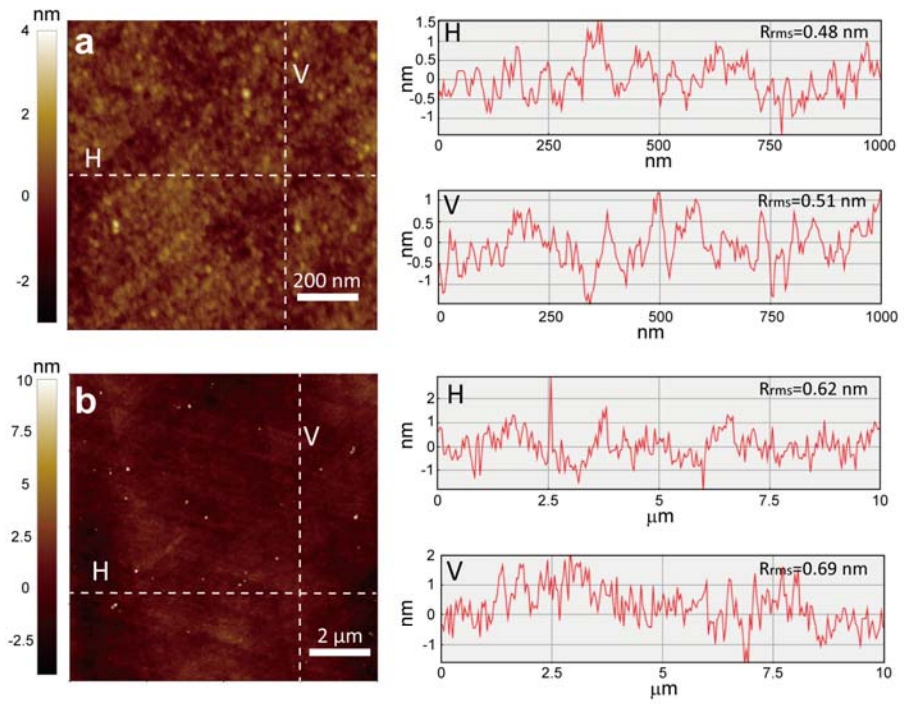

Scanning area		1	2	3	4	5	Avg
1 μm x 1 μm	Horizontal line profiles	0.48	0.51	0.61	0.46	0.43	0.49 ± 0.07
	Vertical line profiles	0.50	0.47	0.50	0.51	0.47	0.48 ± 0.02
10 μm x 10 μm	Horizontal line profiles	1.08	0.95	0.98	0.62	0.66	0.86 ± 0.20
	Vertical line profiles	1.00	0.78	1.06	0.69	0.83	0.87 ± 0.15

Supplementary Figure 1 | AFM measurements on the silver crystal. (**a, b**) Two scanning areas: 1 × 1 μm^2 and 10 × 10 μm^2. The table shows the average root-mean-square (RMS) roughness is 0.5 nm and 0.9 nm, respectively.

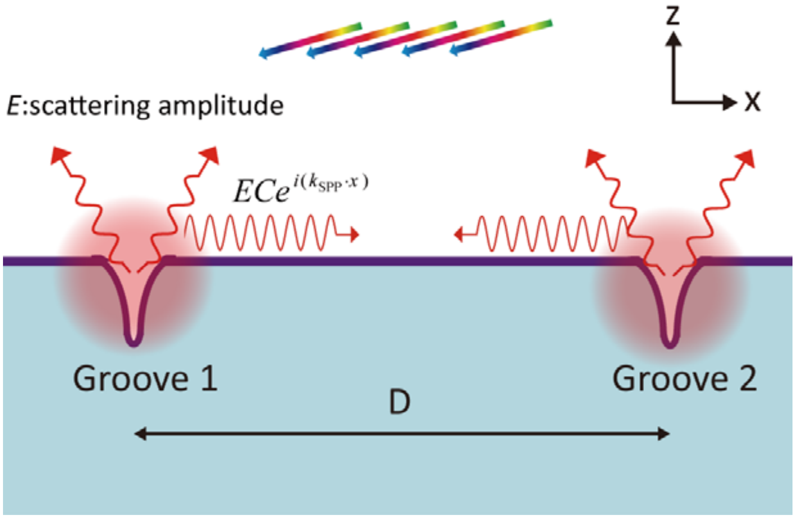

Supplementary Figure 2 | Schematic of SPP interference on the Ag crystal surface. The Ag surface is pattered with a double-groove structure by FIB milling.

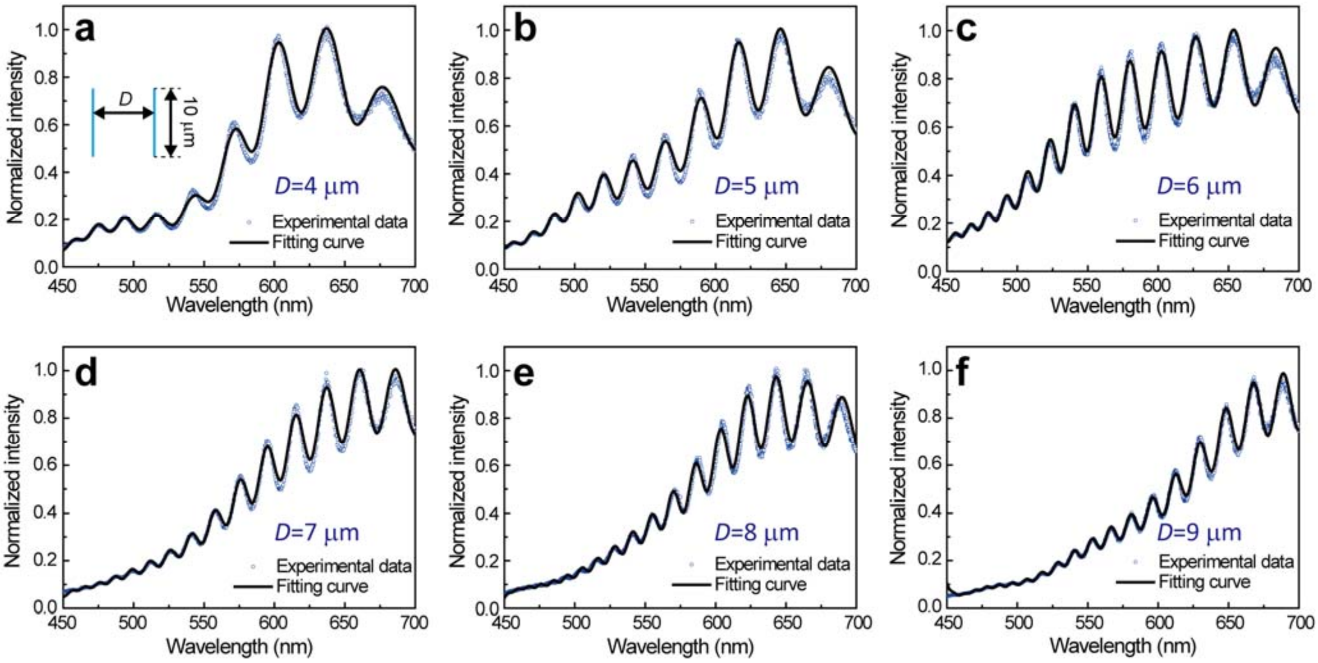

Supplementary Figure 3 | Scattering spectra of double-groove structures with different groove separations. (**a**)-(**f**) SPP Fabry–Pérot interference patterns from double-nanogroove structures with different groove separations. The numbers of intensity oscillations in the displayed spectra window (450–700 nm) increases with increasing *D*, which is consistent with the behavior of a Fabry–Pérot interferometer. Our model can fit very well the experimental spectra from all structures with different groove separations (*D*).

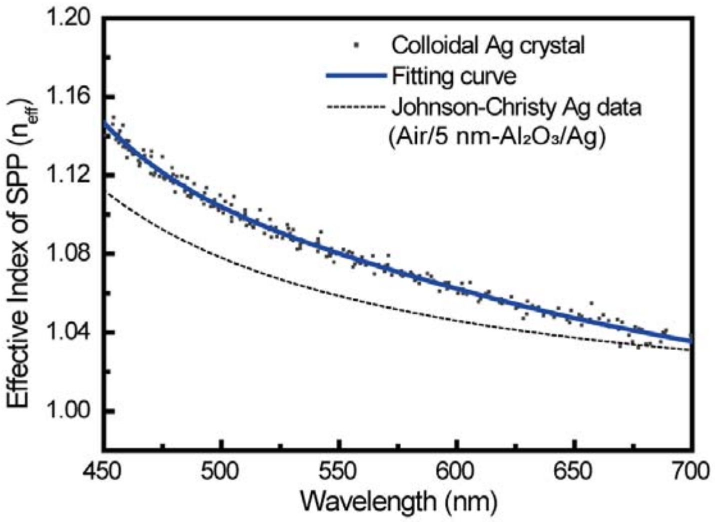

Supplementary Figure 4 | SPP effective index of colloidal Ag single crystal. The resulting effective index extracted from Eq. (S5) in Supplementary Note 2 is self-consistent for all fitted curves shown in Supplementary Fig. 3. In comparison, the black curve is the simulated effective index using the one-dimensional eigenmode method and the Johnson-Christy Ag data (ref. 21).

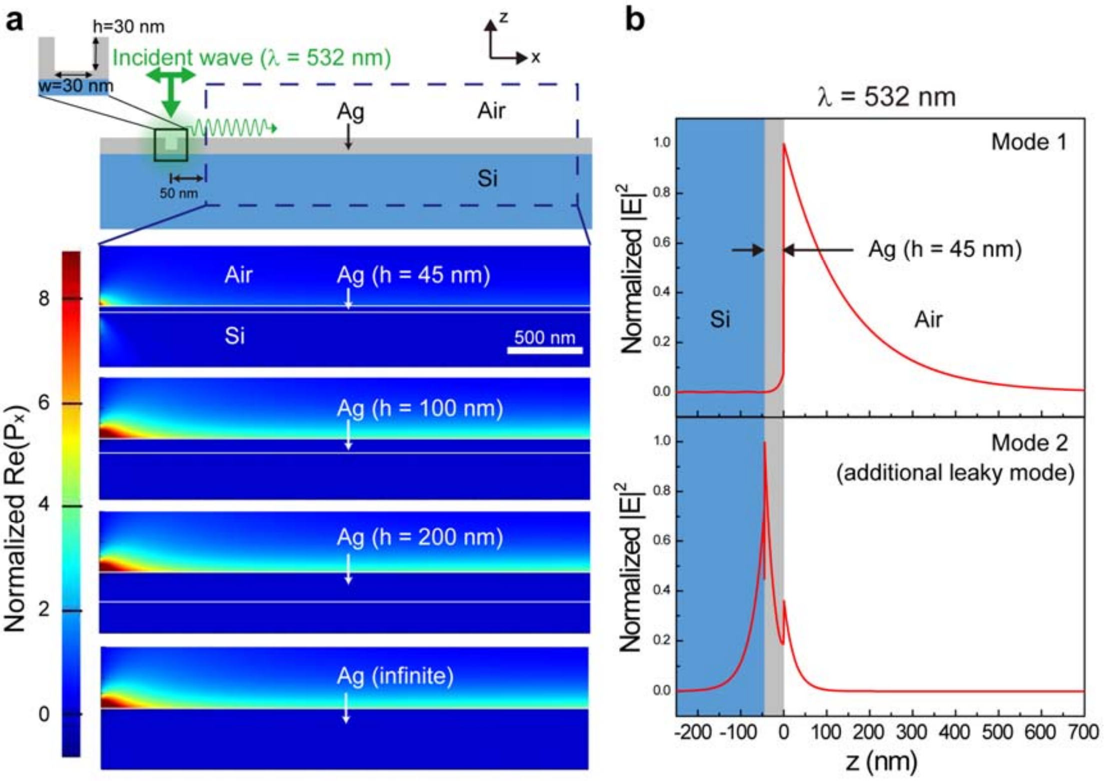

Supplementary Figure 5 | SPP propagation with different silver thicknesses. (**a**) Simulated *x* component of SPP Poynting vector with different Ag thicknesses (45 nm, 100 nm, 200 nm, and infinite thickness) using the FDTD method. The SPP decays faster on a 45-nm-thick silver film than on thicker films (**b**) SPP modes on a 45-nm-thickness silver film using the eigenmode method. The permittivity function of silver is adopted from ref. 16.

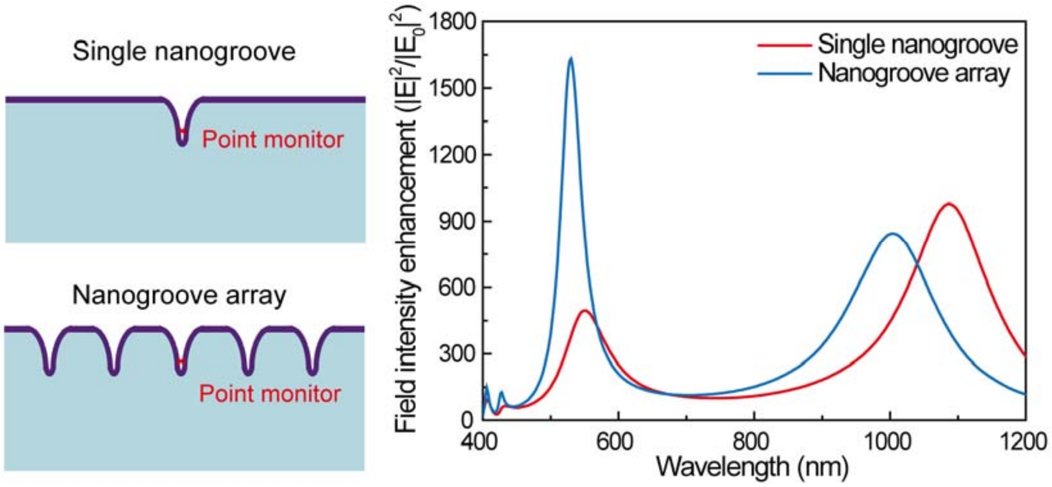

Supplementary Figure 6 | FDTD simulations of field intensity enhancement in nanogroove array structure. The nanogroove array (blue curve) with a 250 nm pitch can increase the field intensity at the SHG wavelength, in comparison to a single nanogroove (red curve). The resonance wavelengths of a nanogroove array are blue shifted with respect to those from a single groove because of the plasmonic coupling effect.

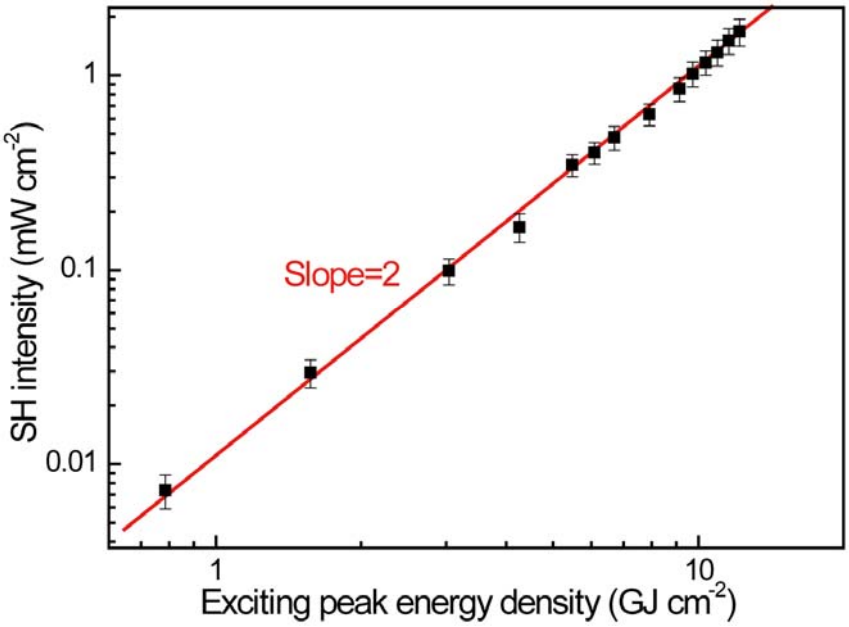

Supplementary Figure 7 | Nonlinear response from nanogroove array structure. Experimental second-harmonic (SH) intensity vs. incident peak energy density plot clearly show the quadratic dependence. The excitation source is a 1064 nm pulsed laser with a 300 fs pulse width and a repetition rate of 40 MHz.

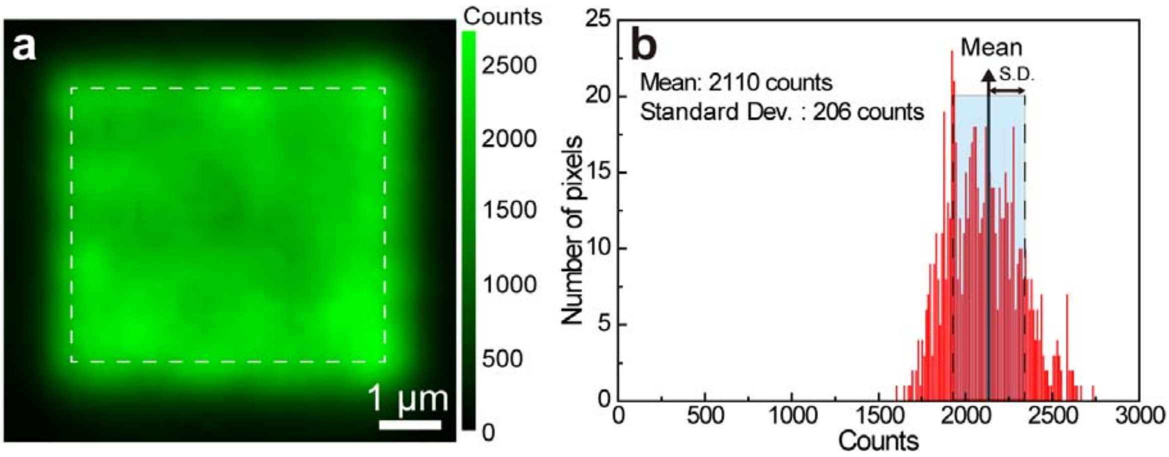

Supplementary Figure 8 | Spatial uniformity of SH emission. (**a**) OM image of spatially uniform SH emission (532 nm) mapped by scanning a focused laser beam (the same one as shown in Fig. 3e). (**b**) Histogram of SH emission intensity, which are acquired from the marked area within dotted lines, as shown in **a**.

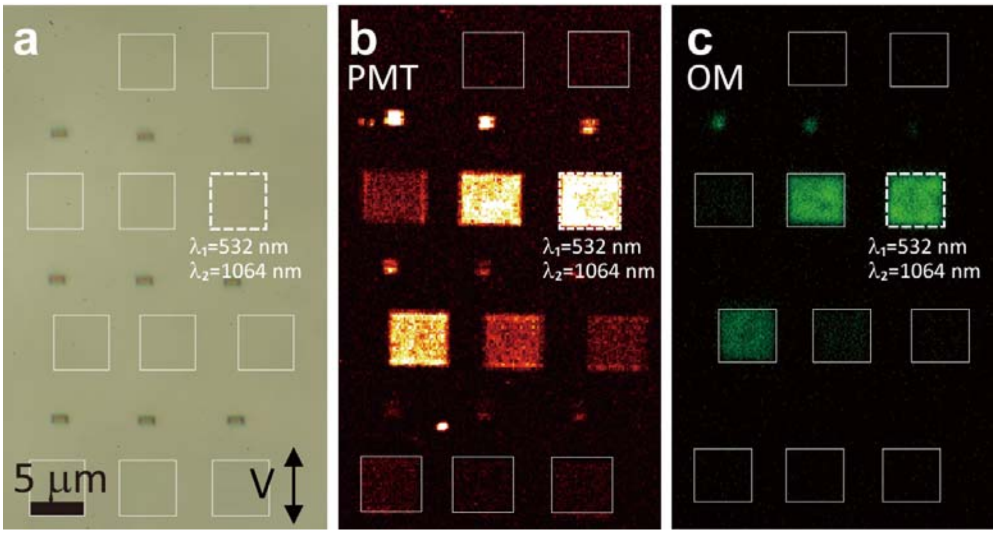

Supplementary Figure 9 | SHG responses in a series of double resonant structures. (**a**) Reflection OM images with a V-polarized incident light. It is consistent with FDTD simulations that the V-polarized incident light cannot excite the plasmonic modes in nanogrooves. (**b, c**) Photomultiplier tube (PMT) and OM images of the SHG signal (532 nm) mapped by using a focused 1064 nm pulsed laser. The excitation laser is polarized perpendicular to the nanogrooves (H-polarized).

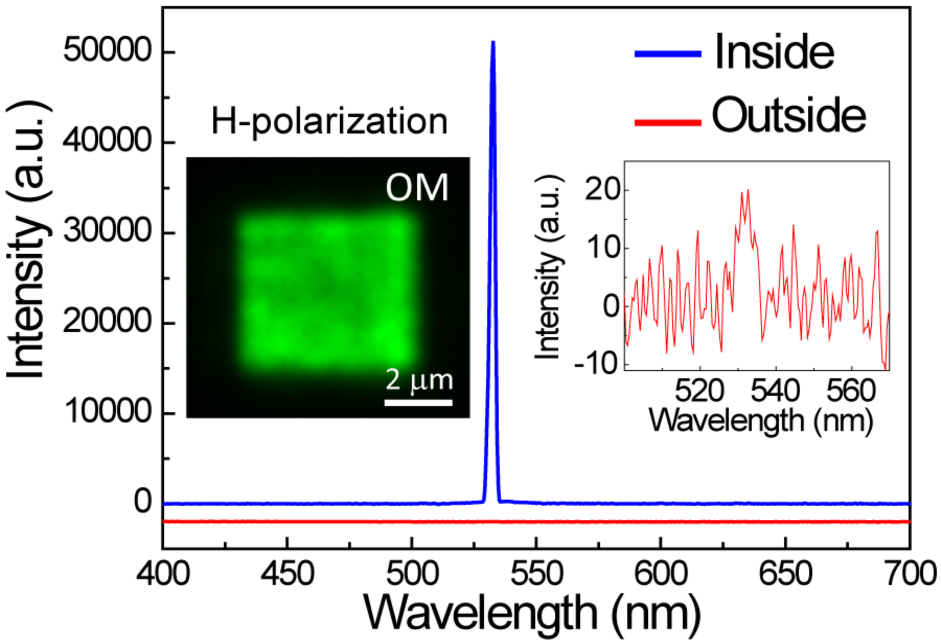

Supplementary Figure 10 | SH enhancement in comparison with an unpatterned surface. The double-resonance design allows both the fundamental and second harmonic fields to be greatly enhanced, leading to more than 2,000 times stronger SH emission in comparison to that from an unpatterned silver surface.

Supplementary Note 1. Atomic force microscopy (AFM)

AFM images (see Supplementary Fig. 1) were acquired by a commercial AFM system (Park system XE-100). The table in Supplementary Fig. 1 shows the root-mean-square (RMS) roughness values from different horizontal and vertical line profiles. The RMS roughness is about 0.5 nm over 1 × 1 μm^2 and 0.9 nm over 10 × 10 μm^2.

Supplementary Note 2. Optical measurements for the determination of SPP propagation lengths at different wavelengths

a. White light interference (WLI, Method 1)

Our objective is to extract the propagation lengths of SPP on the Ag crystal using the Fabry–Pérot (FP) interference patterns resulting from multiple SPP reflections between two grooves, as illustrated in Supplementary Fig. 2. Because of the incoherent nature of the excitation source and the oblique incident angle, the SPP intensity launched from groove 1 is stronger. Therefore, in the following discussion, we only consider SPPs launched from groove 1, reflecting back from groove 2 and subsequent multiple reflections between two grooves. The SPPs propagating along the +*x*-direction on the Ag surface can be describe by

$$E \cdot Ce^{i(k_{\mathrm{SPP}} \cdot x)}, \tag{S1}$$

where E is the amplitude of the detected scattering light from one groove, and E follows the plasmonic resonance of a single nanogroove. The coefficient C is the SPP coupling coefficient, and $k_{\mathrm{SPP}}(\lambda)=k_{\mathrm{r}}(\lambda)+ik_{\mathrm{i}}(\lambda)$ is the SPP wavenumber. The real part of k_{SPP} (k_{r}) determines the peak and dip positions of the interference pattern, and the imaginary part of k_{SPP} (k_{i}) determines the propagation length of SPP such that $L_{\mathrm{SPP}} = 1/(2k_{\mathrm{i}})$.

The scattered light amplitude arising from the FP modes formed between two grooves separated by a distance D can be described as the following,

$$\begin{aligned} A &= E + ECe^{-k_{\mathrm{r}}D}e^{-k_{\mathrm{i}}D}(-r)e^{ik_{\mathrm{r}}D}e^{-k_{\mathrm{i}}D} + ECe^{2ik_{\mathrm{r}}D}e^{-2k_{\mathrm{i}}D}(-r)(-r)^2e^{2ik_{\mathrm{r}}D}e^{-2k_{\mathrm{i}}D} + \ldots \\ &= E + EC(-r)e^{2ik_{\mathrm{r}}D}e^{-2k_{\mathrm{i}}D}(1 + r^2e^{2ik_{\mathrm{r}}D}e^{-2k_{\mathrm{i}}D} + \ldots) \\ &= E - ECre^{2ik_{\mathrm{r}}D}e^{-2k_{\mathrm{i}}D}K \end{aligned}, \tag{S2}$$

$$\text{where}\quad K=\frac{1}{1-r^2e^{2ik_\mathrm{r}D}e^{-2k_\mathrm{i}D}}$$

and the r is the reflection coefficient, which is proportional to the plasmonic resonance spectral shape and the SPP reflection cross-section. Then, the scattering intensity from groove 1 can be written as

$$I\approx|A|^2 . \tag{S3}$$

We first extract $k_\mathrm{r}(\lambda)$, which only depends on the peak and dip positions of the scattering spectrum (Fig. S3a). The precise values of E, C, r, and k_i do not affect the fitted values of $k_\mathrm{r}(\lambda)$. We can simplify them by assuming $E = C = r = 1$, and $e^{-2k_\mathrm{i}D}=0.1$ such that

$$A=1-0.1\frac{e^{2ik_\mathrm{r}D}}{1-0.1e^{2ik_\mathrm{r}D}} . \tag{S4}$$

According to Eq. (S4), the peaks and dips satisfy the following conditions:

$$\begin{aligned}&\text{dips: } 2k_\mathrm{r}D=2q\pi \quad (q=0,\ 1,\ 2,\ ....)\\ &\text{peaks: } 2k_\mathrm{r}D=(2q+1)\pi \quad (q=0,\ 1,\ 2,\ ....)\cdot\end{aligned} \tag{S5}$$

We can then extract the $k_\mathrm{r}(\lambda)$ from Eq. (S5). After that, we substitute the k_r into Eq. (S3) to fit the interference pattern shown in Fig. 2c to extract $k_\mathrm{i}(\lambda)$. To extract k_i one needs to simulate the field distribution and spectral response of SPP launched from the groove, which have been included in the reflection coefficient r. Furthermore, we also use this model to fit interference patterns formed between several double-grooves with different separation distances (see Supplementary Fig. 3) and the resulting effective index ($n_\mathrm{eff} = \lambda/(2\pi)\cdot k_\mathrm{r}(\lambda)$) is self-consistent for all fitted curves (see Supplementary Fig. 4). The SPP propagation distance ($L_\mathrm{SPP}(\lambda)=1/(2k_\mathrm{i}(\lambda))$) shown in Fig. 2e is the fitted value averaged from several double-groove structures with different separations.

b. Direct scattering intensity (DSI, Method 2)

For DSI experiments, the incident laser was focused to a spot size of 10 μm in diameter onto the short launching grooves via a lens of focal length 18 mm. The decoupled light from the long output groove was collected by a 50× long working distance objective. The incident light was polarized at 45 degree relative to the groove. A linear polarizer at –45 degree was used to remove the background from incident light in the

output path. The far-field intensity of scattering light was obtained from CCD images of the output coupler after subtracting a background. Signal from an area of 10 μm (perpendicular to output groove) by 45 μm (parallel to output groove) was used to obtain the integrated output intensity.

c. Film thickness effect in SPP propagation length

The silver thickness would affect the SPP propagation length only when the thickness is below 100 nm. We use the FDTD method to simulate SPP propagation lengths on Ag films with different thicknesses (45 nm, 100 nm, 200 nm and infinite thickness, as shown in Supplementary Fig. 5a). In these simulations, an incident light with λ = 532 nm excites a single nanogroove on the silver film to generate SPPs. The propagation length on a 45-nm-thick silver film is clearly shorter than that in other films (see Supplementary Fig. 5a) because of the presence of an additional leaky mode (mode 2) in Supplementary Fig. 5b. The propagation length no longer depends on film thickness when it exceeds 100 nm (see Supplementary Fig. 5a).

Supplementary Note 3. SHG measurement

a. Field distribution of fundamental plasmonic resonance

We used the two-dimensional FDTD method to simulate the near-field distributions at 532 nm and 1064 nm (see Fig. 3d and Supplementary Fig. 6). The permittivity function of silver is adopted from ref. 16.

b. Optical setup of reflectance measurement

We used a halogen white light as the incident light to excite the nanogroove array at normal incidence through a 100× objective lens (Olympus, N.A. = 0.8) and a polarizer. The reflection light was collected by the same objective lens. All the reflectance spectra shown in Figs. 3c and 4d are normalized to the reflection spectrum from a bare silver single crystal.

c. Optical setup for SHG measurement

We used a homemade confocal microscope to map the SHG signal by scanning the sample under the

excitation laser spot. The signal was projected onto a photomultiplier tube (PMT) and a charge-coupled device (Andor, iDus DU420A-BU2), which was cooled to –60˚C. A short-pass filter (Semrock, FF750-SDi02) separated the SHG signal from the excitation light. The excitation source is a 1064 nm pulsed laser (Fianium, FemtoPower 1060-532-s) with a 300 fs pulse width and a repetition rate of 40 MHz. The optical excitation and collection of the SHG signal from the nanogroove arrays were through the same 100× objective lens (Nikon, N.A. = 0.9) at normal incidence/collection geometry. Furthermore, the spot diameter is 1.4 μm.

d. More detailed descriptions for Supplementary Figs. 7, 8, 9 and 10

In Supplementary Figure 7, we show the quadratic relationship between the SH signal and the incident excitation peak energy density in a log–log plot. The SHG conversion efficiency ($P^{(2\omega)}/p^{(\omega)}$) depends on various conditions including the excitation pulse, the focused spot size and excitation cross-section, thus difficult to compare between different structures. With an incident average power of 2.4 mW, we measured a value of 1.2×10^{-8}, which is comparable with other plasmonic nanostructures[30]. We note that this SHG efficiency is limited by the nonlinear coefficient of Ag itself. A higher efficiency can be achieved by placing other dielectric nonlinear materials in plasmonic hot spots or hot areas. In Supplementary Figure 8, we show the histogram of SH emission from a square area (5×5 μm^2). This statistical analysis shows that the standard deviation of SH intensity is about 10% of the mean value. In Supplementary Figure 9, we provide more data about tunable plasmonic resonant structures for enhanced SHG (Fig. 4). In comparison to the H-polarized incident light, the V-polarized incident light (i.e., E field parallel to the nanogroove) cannot excited the plasmonic modes in nanogrooves; thus, no reflection color change can be observed in Supplementary Fig. 9a. The SHG signal is spatially uniform as shown in Supplementary Fig. 9b,c. Supplementary Figure 10 shows that the double resonant structure allows more than 2,000 times stronger SH emission, in comparison with that from an unpatterned surface.
